# Role of the DNA damage response in prostate cancer formation, progression and treatment

**DOI:** 10.1038/s41391-019-0153-2

**Published:** 2019-06-13

**Authors:** Wenhao Zhang, Dik C. van Gent, Luca Incrocci, Wytske M. van Weerden, Julie Nonnekens

**Affiliations:** 1 Department of Molecular Genetics, Erasmus MC, Rotterdam, The Netherlands; 2 Oncode Institute, Erasmus MC, Rotterdam, The Netherlands; 3 Department of Radiation Oncology, Erasmus MC Cancer Institute, Rotterdam, The Netherlands; 4 Department of Experimental Urology, Erasmus MC, Rotterdam, The Netherlands; 5 Department of Radiology and Nuclear Medicine, Erasmus MC, Rotterdam, The Netherlands

**Keywords:** Cancer therapy, Cancer genetics, Cancer genetics, Cancer therapy

## Abstract

**Background:**

Clinical and preclinical studies have revealed that alterations in DNA damage response (DDR) pathways may play an important role in prostate cancer (PCa) etiology and progression. These alterations can influence PCa responses to radiotherapy and anti-androgen treatment. The identification of DNA repair gene aberrations in PCa has driven the interest for further evaluation whether these genetic changes may serve as biomarkers for patient stratification.

**Methods:**

In this review, we summarize the current knowledge on DDR alterations in PCa, their potential impact on clinical interventions and prospects for improved management of PCa. We particularly focus on the influence of DDR gene mutations on PCa initiation and progression and describe the underlying mechanisms.

**Results and Conclusions:**

A better understanding of these mechanisms, will contribute to better disease management as treatment strategies can be chosen based on the specific disease properties, since a growing number of treatments are targeting DDR pathway alterations (such as Poly(ADP-ribose) polymerase inhibitors). Furthermore, the recently discovered crosstalk between the DDR and androgen receptor signaling opens a new array of possible strategies to optimize treatment combinations. We discuss how these recent and ongoing studies will help to improve diagnostic, prognostic and therapeutic approaches for PCa management.

## Introduction

Prostate cancer (PCa) is the second most common cancer in men and the fourth most common tumor type worldwide [1]. Although organ-confined disease can be well managed, curative therapeutic options for disseminated disease are limited. First-line therapy for disseminated PCa is androgen deprivation therapy (ADT) that prevents androgen receptor (AR) pathway signaling as most PCas are dependent on activated AR signaling for cell survival [2, 3]. In time, patients under ADT may progress to castration-resistant PCa (CRPC), requiring first line chemotherapy (commonly docetaxel) [4]. New therapeutic strategies for CRPC are being offered to patients, such as new combinations and sequences of second-generation antiandrogen therapy (enzalutamide, abiraterone, apalutamide) or second line chemotherapy (cabazitaxel), which have shown notable benefit for patient survival [4]. In addition, promising new treatment modalities, such as Radium-223 and prostate-specific membrane antigen (PSMA)-directed radioligand therapy, are being exploited for patients with (bone) metastatic disease. Despite this progress in the development of new drugs, CRPC continues to be incurable, and drug resistance remains an issue.

Clinical and preclinical studies have revealed that alterations in DNA damage response (DDR) pathways play a role in PCa etiology and progression, especially in CRPC patients [5–10]. These DNA repair defects may be targeted by specific treatments, such as Poly(ADP-ribose) polymerase (PARP) inhibitors [11]. Moreover, several studies provided evidence that AR signaling links to the DDR in prostate cancer cells, which may have relevance for the first line disease management using ADT and AR-targeted agents [12, 13]. In this review, we summarize the current knowledge of DDR alterations in PCa, the AR-DDR crosstalk and the potential exploitation of DDR targeting drugs to improve clinical interventions.

## DNA damage response pathways

DNA damage has emerged as a major culprit in cancer initiation and progression. The DNA is constantly damaged by exogenous sources such as genotoxic chemicals, ultraviolet (UV), and ionizing radiation (IR), as well as by endogenous DNA-damaging agents, such as reactive oxygen and nitrogen species [14, 15]. These sources will induce various damages to the DNA, including base oxidation, deamination, alkylation, interstrand crosslinks, adduct formation, single-strand breaks (SSBs), and double-strand breaks (DSBs). Additionally, spontaneous DNA damage is induced during replication. Collisions of the replication fork with DNA-binding proteins or the transcription machinery are the most common causes leading to replication fork stalling or collapse, which in turn induces DNA damage [16, 17]. Incorrect or failed repair of damaged DNA can lead to genetic alterations. Important consequences of genetic alterations are loss of tumor suppressor genes and activation of oncogenes, which may trigger the development of malignant cells or increase aggressiveness of tumor cells. Normal cells maintain genomic integrity using various DDR mechanisms to repair damaged DNA or induce cell death. The concept of DDR has been introduced to describe a series of biological reactions including DNA lesion site detection, repair protein recruitment, damage repair, cell cycle checkpoint control, and cell death pathways.

**Fig. 1:**
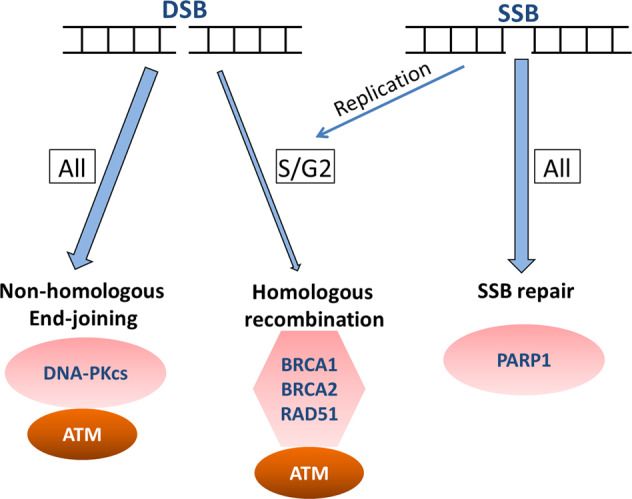
DNA double strand (DSB) and single-strand break (SSB) repair pathways. The majority of the DSBs are repaired by the error-prone Non-Homologous End-Joining pathway (NHEJ, available during all cell cycle stages) and a smaller fraction of the DSBs are repaired via Homologous Recombination (HR, only during S/G2 cell cycle stages). SSBs are repaired by the Single Strand Break Repair pathway (available during all cell cycle stages). During DNA replication an unrepaired SSB can  be converted into a DSB which can then only be repaired by HR

The highly diverse spectrum of DNA lesions can be repaired by a number of different DNA repair pathways, which have been reviewed extensively elsewhere [18–20]. In short, base excision repair (BER) involves multiple enzymes to excise and replace a single damaged nucleotide base, such as an oxidized base, but also an SSB [21]. Mismatch repair (MMR) is mainly involved in repair of base mismatches and insertions/deletions that can occur during replication and recombination [22]. The Fanconi anemia (FA) pathway repairs DNA interstrand crosslinks in the genome [23]. DSBs are resolved either by high-fidelity homologous recombination (HR) or error-prone non-homologous end joining (NHEJ). The HR pathway is only active when the cell is in the S/G2 cell cycle stage since it requires the presence of the sister chromatid as a repair template [24]. DSBs can be generated during replication when the replication fork encounters a DNA lesion and these breaks are exclusively repaired by HR. NHEJ is active during all cell cycle stages and functions by directly ligating broken DNA ends. Since no template is used during NHEJ, repair via this pathway is error prone (Fig. 1) [24]. After DNA damage induction, depending on the severity of the lesion and repair capacity, cells will continue to proliferate if damages are repaired, or cells stop proliferation, become senescent, or undergo programmed cell death (apoptosis) to remove damaged DNA from the cellular population [25]. Alterations in any of these pathways can result in genomic instability and consequently predispose to cancer, affect disease progression and/or influence therapy efficacy. Nonetheless, impaired DNA repair can also be a possible Achilles heel of the cancer that can be exploited for treatment [26].

## Molecular mechanisms underlying PCa risk

Multiple studies have indicated that germline mutations in DNA repair genes are associated with a higher risk of developing PCa. The individuals at risk have one inherited dysfunctional allele of the DNA repair gene and a second event (mutation or epigenetic silencing) can cause inactivation of the functional allele. The most common germline mutated DDR genes in primary PCa or CRPC are found in the Breast Cancer 1 and 2 (*BRCA1* and *BRCA2*) genes. Similar to the role of mutations in *BRCA1/2* in the development of breast cancer and ovarian cancer [14], various studies have shown that inactivating *BRCA1/2* mutations, predominantly *BRCA2*, increase predisposition to PCa (Table 1) [7–9, 27–29]. *BRCA1/2* are tumor suppressor genes and both encode large proteins which act in multiple cellular pathways. BRCA1 and BRCA2 are both involved in the HR pathway [30, 31], while BRCA1 has also been found to have other functions [32]. Loss-of-function mutations in *BRCA1/2* lead to a deficiency in error-free HR repair. Therefore, DSBs will be repaired alternatively by other non-conservative and potentially mutagenic mechanisms, such as the NHEJ pathway. The resulting genomic instability (chromosomal translocations and deletions) and mutations may be the underlying mechanism of *BRCA1/2* associated cancers [33, 34]. This could increase the risk of acquiring fusion genes, such as the TMPRSS2/ERG fusion that is found in 40-50% of PCa cases [35], although no solid evidence has been acquired to link *BRCA1/2* mutation status to this fusion. Furthermore, the reason why *BRCA1/2* mutations are particularly associated with specific cancer types, such as breast, ovarian and PCa remains unknown.

**Table 1:** Germline DDR mutations increase PCa risk

Gene	Pathway	Relevance
*BRCA1* [9]	HR	Deleterious *BRCA1* mutations confer a relative PCa risk of 3.75, and a 8.6% cumulative risk by age 65.
*BRCA1* and *BRCA2* [28, 49, 61]	HR	*BRCA2* mutation carriers have an increased risk of PCa and a higher histological grade. *BRCA1* and *BRCA2* mutation carriers had a higher risk of recurrence and PCa-specific death.
*MSH2*, *MLH1*, and *MSH6* [27]	MMR	Increased PCa risk. Evidence to link PCa to Lynch syndrome.
*MLH1*, *MSH2*, *MSH6*, and *PMS2* [7]	MMR	MMR genes may confer a high risk of PCa when mutated.
*MSH2*, *MLH1*, and *MSH6* [29]	MMR	MMR gene mutation carriers have at least a twofold or greater increased risk of developing MMR-deficient PCa where the risk is highest for MSH2 mutation carriers.
*BRIP1* [8]	FA	Truncating mutations in BRIP1 might confer an increased risk of PCa

*BRCA1/2*: Breast Cancer 1 and 2, *MSH2/6*: MutS protein homolog 2 and 6, *MLH1*: MutL homolog 1, *PMS2*: PMS1 homolog 2, *BRIP1*: BRCA1 interacting protein C-terminal helicase1, *HR*: homologous recombination, *MMR*: mismatch repair, *FA*: fanconi anemia pathway

Francis et al. showed that BRCA2 can act as a tumor suppressor in the prostate [36]. Using a genetically engineered mouse model, it was found that deletion of *Brca2* in prostate epithelia resulted in focal hyperplasia and low-grade prostate intraepithelial neoplasia (PIN) in animals over 12 months old. Epithelial cells in these lesions showed an increase in DNA damage. The evidence that other inherited gene mutations in DSB repair genes, such as BRCA1 Interacting Protein C-Terminal Helicase 1 (*BRIP1*) and Nibrin (*NBS1*), are also associated with PCa has been documented less extensively [6, 8].

Besides DSB gene alterations, mutations in the MMR genes MutS homolog 2 and 6 (*MSH2* and *MSH6*) are also associated with increased PCa risk [7, 27]. MMR mutations would mainly cause point mutations or small insertions and deletions of short repetitive sequences of DNA which may result in microsatellite instability [37]. Therefore, underlying mechanisms of PCa can be linked to Lynch syndrome, a hereditary ‘non-polyposis’-colorectal carcinoma that is caused by MMR pathway mutations. The increased risk of PCa in MMR mutation carriers and in families with Lynch syndrome provide the rationale to include PCa in the Lynch syndrome tumor spectrum, which is relevant for risk estimates and surveillance recommendations in MMR mutation carriers [38].

## DDR defects in PCa

### DDR defects in primary PCa

The clinical behavior of localized PCa is highly variable: while some men have aggressive cancer leading to metastasis and death, many others have indolent cancers and these men can be cured by local therapy or may be safely observed without treatment [39]. Several studies have identified primary PCa tumors harboring a diversity of DDR gene alterations (summarized in Table 2) [40–45]. These studies identified a heterogeneous panel of repair defects caused by homozygous mutations or copy number alterations in primary prostate tumors compared to paired normal tissue in Ataxia–telangiectasia mutated (*ATM*), *BRCA2, RAD51*, mediator of DNA damage checkpoint 1 (*MDC1), PARP1*, and FA complementation group D2 (*FANCD2*), although the level of incidence varied between the studies. This considerable heterogeneity of repair defect prevalence among different studies could at least in part be attributed to the diversity of the study populations, as the genetic background can differ significantly between indolent, non-symptomatic and progressive PCa [46–48].

**Table 2:** Prevalence of selected DDR gene alteration in primary PCa

	DDR pathway involved	Barbieri et al. [41]	Baca et al. [40]	Cancer Genome Atlas [42]	Fraser et al. [45]	Ren et al. [44]	Total
Number of patients		112	57	333	449	65	1017
ATM	General	2.8% (3)	12.5% (7)	7.2% (24)	1.8% (8)	3.1% (2)	4.3% (44)
ATR			1.8% (1)	2.4% (8)		5% (3)	1.2% (12)
BRCA1	HR	1.8% (2)		1.2% (4)		1.5% (1)	0.69% (7)
BRCA2			7.1% (4)	3.3% (11)		1.5% (1)	1.58% (16)
RAD51			3.6% (2)	2.1% (7)			0.88% (9)
PARP1	BER		3.6% (2)	3.0% (10)		3.1% (2)	1.38% (14)
MLH1	MMR			0.3% (1)			0.09% (1)
MSH2				1.5% (5)			0.49% (5)
FANCD2	FA		1.8% (1)	0.9% (3)		1.5% (1)	0.49% (5)
All genes		4.6%	30.4%	21.9%	1.8%	15.7%	11.1%

Data was acquired from The Memorial Sloan Kettering cBioportal database (http://cbioportal.org)

*ATM*: ataxia–telangiectasia mutated serine/threonine kinase, *ATR*: ATM and RAD3-related serine/threonine kinase, *BRCA1/2*: Breast Cancer 1 and 2, *RAD51*: RAD51 recombinase*, PARP1*: poly(ADP-ribose) polymerase 1, *MLH1*: MutL homolog 1, *MSH2*: MutS protein homolog 2, *FANCD2*: FA complementation group D2, *HR*: homologous recombination, *BER*: base excision repair, *MMR*: mismatch repair, *FA*: fanconi anemia pathway

Loss-of-function DDR gene mutations can contribute to a more aggressive PCa phenotype with a higher probability of nodal involvement and distant metastasis [5, 49–51]. This aggressive phenotype was also reported in patients harboring *BRCA1/2* and *ATM* combined mutations [52] and *NBS1* mutations alone [6]. Recent clinical data have shown a strong prognostic value of a DDR mutation signature which may be used for risk stratification for high-risk PCa patients. Treatment outcome for *BRCA1/2* mutation carriers showed worse outcomes for these patients than non-carriers when conventionally treated with surgery or radiation therapy [53].

The studies discussed above found DDR mutations in primary PCa, with a heterogeneous and overall low mutation rate. However, a direct (mechanistic) link between these mutations and PCa predisposition and treatment has not yet been established. As primary PCa is typically well managed and not lethal, it will therefore be of more interest to focus on the landscape of DDR defects in advanced PCa.

### DDR defects in mCRPC

An enrichment of DDR gene alterations can be found during PCa progression, especially when the disease develops into metastatic CRPC (mCRPC) (summarized in Table 3) [54–56]. Heavily pre-treated mCRPC contained more genetic alterations in DDR genes (46%) than treatment-naive high grade localized tumors (27%) [54]. A multi-institutional clinical sequencing study revealed that the majority of affected individuals with CRPC harbor clinically actionable homozygous molecular alterations, with 23% of mCRPC harboring DDR aberrations and 8% harboring DDR germline mutations [55]. Aberrations in *BRCA1, BRCA2*, and *ATM* were observed at substantially higher frequencies (19.3% overall) in mCRPC compared to those in primary PCa. Among these DDR alterations, *BRCA2* was the most frequently altered (12.7%), and ∼90% of these *BRCA2* defective tumors exhibited biallelic loss. As aberrations in these genes are expected to confer sensitivity to PARP inhibitors [56], nearly 20% of mCRPC patients may potentially benefit from this therapy. Additionally, three out of four mCRPC tumors in this cohort which presented hypermutations are harboring defects in the MMR pathway genes MLH1 or MSH2 [55]. Whether this abundance of DDR alterations is specifically targeted to these genes or a general consequence of high mutational burden for advanced disease is still unclear.

**Table 3:** Prevalence of selected DDR genes alteration in mCRPC

	DDR pathway involved	Grasso et al. [54]	Robinson et al. [55]	Total
Number of patients		59	150	209
ATM	General	11.8% (7)	5.3% (8)	7.2% (15)
ATR		5% (3)	8.6% (13)	7.7% (16)
BRCA1	HR		0.7% (1)	0.5% (1)
BRCA2		11.8% (7)	9.3% (14)	10.0% (21)
RAD51		1.7% (1)	2.0% (3)	1.9% (4)
PARP1	BER	3% (2)	2.7% (4)	5.5% (6)
MLH1	MMR	1.7% (1)	1.3% (2)	1.4% (3)
MSH2		3.3% (2)	2.7% (4)	2.9% (6)
FANCD2	FA	3.3% (2)	2.7% (4)	2.9% (6)
All genes		41.6%	35.3%	40%

Data was acquired from The Memorial Sloan Kettering cBioportal database (http://cbioportal.org)

*ATM*: ataxia–telangiectasia mutated serine/threonine kinase, *ATR*: ATM and RAD3-related serine/threonine kinase, *BRCA1/2*: Breast Cancer 1 and 2, *RAD51*: RAD51 recombinase, *PARP1*: poly(ADP-ribose) polymerase 1, *MLH1*: MutL homolog 1, *MSH2*: MutS protein homolog 2, *FANCD2*: FA complementation group D2, *HR*: homologous recombination, *BER*: base excision repair, *MMR*: mismatch repair, *FA*: fanconi anemia pathway

### DDR defects and response to PCa treatment

Various retrospective and prospective studies have been performed in which treatment outcome to conventional PCa treatment was compared in DDR mutation carriers and wild-type individuals. The prognostic and predictive impact related to standard therapies for DDR mutated mCRPC has yet to be determined, since these trials (summarized in Table 4) report inconsistent and conflicting outcomes: one study found no difference between the patient groups [57], while other studies reported DDR mutation carriers to have either inferior [58] or improved responses [59, 60] to the therapy. This inconsistency could be explained in several ways. First, the number of mCRPC patients harboring DDR mutations is very limited in each cohort. Second, the results can be biased due to different sampling, as metastatic biopsies are only feasible for patients with low-to-moderate tumor burden. This might exclude highly aggressive tumors and blood-based sequencing may underestimate the mutation rate as the somatic status is unknown for certain patients. Third, the disease showed extensive heterogeneity and patients had received various pre-treatments in the different cohorts. A recent prospective study showed that *BRCA2* mutation carriers have a worse outcome in mCRPC disease and this may be affected by the first line treatment used [61]. However, future prospective studies are needed to shed further light on this issue and will hopefully resolve the above-mentioned controversy.

**Table 4:** Clinical outcome of mCRPC patients with wild type vs DDR gene mutations after standard AR-targeting therapy

Author and year	Study design	Sampling	Treatment	DDR defect patients	PSA-PFS	OS
Annala et al. 2017 [58]	Retrospective four cohorts	Blood germline	Enzalutamide/ Abiraterone	24/319 (7.5%)	3.3 mo DDR(-) vs 6.2 mo WT	29.7 mo DDR(-) vs 34.1 mo WT
Mateo et al. 2018 [57]	Retrospective two cohorts	Blood germline	Enzalutamide/ Abiraterone	60/390 (15.4%)	8.3 mo DDR(-), vs 8.3 mo WT	36 mo DDR(-) vs 38.4 mo WT
Antonarakis et al. 2018 [59]	Retrospective/ prospective Single cohort	Blood germline	Enzalutamide/ Abiraterone	22/172 (12%)	10.2 mo DDR(-) vs 7.6 mo WT	41.1 mo DDR(-) vs 28.3 mo WT
Hussain et al. 2018 [60]	Randomized phase 2 multicenter trial	Biopsy mixed	Abiraterone plus Prednisone	20/80 (25%)	16.6 mo DDR(-) vs 8.2 mo WT	N/A
Castro et al. 2019 [61]	Prospective multicenter/cohort	Blood germline	Abiraterone Enzalutamide	16/302 (5.3%) 8/126 (6.3%)	8.1 mo DDR(-) Vs 9.2 mo WT (combined)	N/A

*PSA*: prostate-specific antigen, *PFS*: progression-free survival, *OS*: overall survival, *DDR*: DNA damage response, *WT*: wild-type

Radium-223, a bone-seeking α-particle emitter that induces DSBs, thereby killing cancer cells in the bone microenvironment, is commonly used for CRPC patients with symptomatic bone metastases [62]. Recently, a retrospective single-institution study showed that germline or somatic HR-deficient patients responded better to Radium-223 therapy compared to wild-type patients, with a better alkaline phosphatase responses (80% vs 39%, *p* = 0.04), and a trend toward longer overall survival (median 36.9 vs 19.0 months, *p* = 0.11) [63]. Synthetic lethality between HR mutations and Radium-223 activity maybe the underlying mechanism of a better efficacy, however these promising results need further (prospective) validation.

## AR and DDR pathway crosstalk

Clinical trials have shown that the combination of ADT or anti-androgens with radiotherapy significantly increases patients survival and reduces distant metastases compared to radiotherapy alone [64–69]. It is widely perceived that suppression of the AR axis enhances the cytotoxic effects of radiotherapy and based on the beneficial effects, this combination is currently the standard of care for locally advanced PCa.

**Fig. 2:**
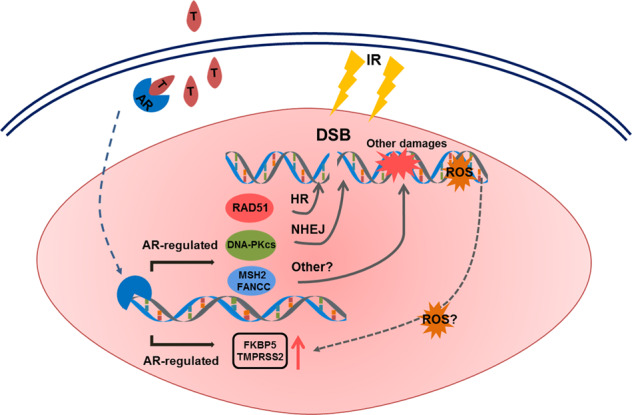
Interplay between androgen receptor (AR) and DNA damage repair in prostate cancer. Activation of AR by dihydrotestosterone (T) leads to transcriptional upregulation of DNA repair genes in various repair pathways. Reciprocally, irradiation results in upregulation of keys genes in the AR pathway via ROS. HR, homologous recombination; NHEJ, non-homologous end-joining; ROS, Reactive oxygen species; IR, Irradiation

The molecular mechanism of radiosensitization induced by ADT was investigated in preclinical studies. Goodwin et al. reported that ADT potentiates the tumor-killing effect of ionizing radiation (IR) in AR proficient cells both in vitro and in vivo: ADT treated C4-2 (androgen independent) cells had a diminished capacity to repair IR induced DSBs. This study showed that the AR pathway directly regulates the NHEJ factor DNA-dependent protein kinase catalytic subunit (DNA-PKcs), resulting in a slight increase in NHEJ activity upon androgen addition in a plasmid-based functional assay [12]. The involvement of NHEJ was confirmed by Polkinghorn et al. who identified a set of 32 DDR genes as direct AR target genes [70]. Other studies using patients samples have demonstrated that castration primarily reduces Ku70 protein expression, which is essential for NHEJ [71, 72]. These studies suggest that ADT enhances IR effects by impairing NHEJ activity. Reciprocally, IR treatment caused marked induction of the androgen target genes *TMPRSS2* and *FKBP5* [12], suggesting that DNA damage induces AR activity (Fig. 2).

In addition to direct regulation of the NHEJ pathway, other studies show that AR signaling plays a role in regulating genes involved in the HR, MMR, and FA pathways [12, 70, 73]. Enzalutamide treatment suppressed the expression of the HR genes *BRCA1, RAD54L*, and RecQ Mediated Genome Instability 2 *(RMI2)* [73]. A combination strategy in which enzalutamide pretreatment was followed by the PARP inhibitor olaparib resulted in significantly increased PCa cell apoptosis and inhibited colony formation in vitro. Further in vivo evaluation showed clear synergistic suppressive effects on PCa xenografts in hormone-sensitive models, but not in CRPC models [73]. However, from these studies, it is not yet clear whether enzalutamide directly induces HR deficiency, also called the BRCAness phenotype. A reduction of the S/G2 cell cycle fraction might also have caused reduction of HR gene expression, which resulted in reduced HR in the total cell population. Whatever the mechanistic explanation may be, this study warrants further clinical investigation into AR and PARP inhibitor combination therapies.

Based on these results, it is clear that both preclinical and clinical studies have found that AR signaling regulates the expression and/or function of DDR genes. Elucidation of the precise regulatory mechanisms and pathway interactions requires additional studies, which should focus on direct measurement of NHEJ and HR capacity in the presence and absence of AR signaling.

## Exploiting DDR alterations for PCa treatment

As discussed above, 10–25% of PCa patients are harboring DDR mutations, especially among mCRPC patients. This section summarizes clinical and preclinical evidence how DDR alterations could be exploited therapeutically.

### Immune checkpoint inhibitors

The successful development of immune checkpoint inhibitors such as programmed cell death protein 1 (PD-1) and programmed death-ligand 1 (PD-L1) inhibitors revolutionized the field of cancer immunotherapy [74]. The interaction of PD-L1 on tumor cells with PD-1 on T-cells reduces T-cell functionality, preventing the immune system from attacking the tumor cells. Inhibitors that block this interaction can unleash a patient’s own T cells to kill tumors [75]. Immunotherapy responses appear to correlate with the mutational burden, presumably by the increase in neo-antigens [76]. PCa patients harboring MMR mutations, such as in *MLH1* or *MSH2*, could be selected for PD-1 blockade immunotherapy, as a favorable response to PD-1 blockade therapy was observed previously in MMR-deficient tumors, as a result of the high level of neo-antigens in various solid tumors [77]. Interestingly, ductal adenocarcinoma, an aggressive histopathology of PCa, is associated with MMR defects, suggesting that these patients are possible candidates for this type of immunotherapy [78]. Interestingly, an increase in neo-antigens was also observed in patients who harbor a HR deficiency [79]. Altogether, these subgroups represent nearly 20% of mCRPC patients, making the use of PD-1/PD-L1 inhibitors a potentially attractive strategy for clinical trials in these patients.

### PARP inhibitor treatments

#### Monotherapy

**Fig. 3:**
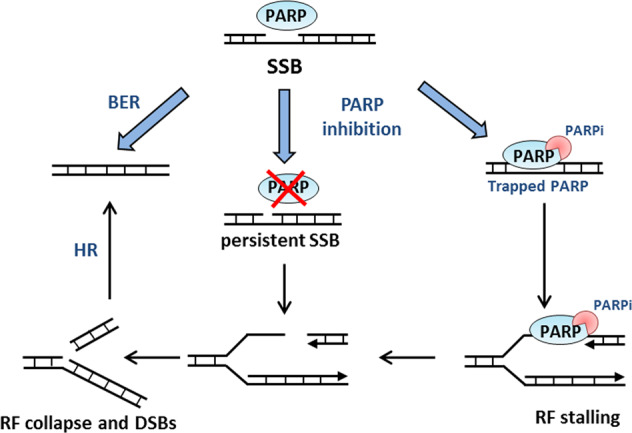
Mechanism of action of Poly(ADP-ribose) polymerase (PARP) inhibitor. PARP enhances repair of single-strand breaks (SSBs) via base excision repair (BER). If SSBs remain unrepaired due to inhibition of PARP catalytic activity with PARP inhibitors (PARPi), double-strand breaks (DSBs) can be formed during replication. Alternatively, PARPi can trap the PARP protein on the DNA, which causes replication fork (RF) stalling and collapse. Homologous recombination (HR) is essential for repairing these DSBs

Tumors with compromised HR are highly sensitive to reduction of SSB repair by PARP1 inhibition, a phenomenon called synthetic lethality [80–82]. The mechanism of action of PARP inhibitors was originally described as inhibition of SSB repair via blocking the catalytic activity of PARP1. Unrepaired SSBs will be converted into the more genotoxic DSBs during DNA replication. These DSBs are repaired via HR in normal cells, but cannot be repaired in HR-deficient cancer cells, leading to tumor-specific cell death. Recently, this model has been updated as studies have shown that various PARP inhibitors are able to trap PARP1 at the DNA damage site [83–85]. Trapped PARP results in DSBs when the replication fork encounters this lesion, which require HR for resolution (Fig. 3). Considering the different PARP trapping abilities of the different PARP inhibitors, various therapeutic responses can be expected, with talazoparib having the most profound PARP trapping and cytotoxic effects [86].

Following previous in vitro [80, 81] and in vivo studies in *Brca2* knockout breast and ovarian tumor mouse models [87, 88], a number of trials evaluated PARP inhibitors as a single agent in CRPC patients with HR defects. The TOPARP study evaluated olaparib in a population of 50 mCRPC patients. Interestingly, 14 out of 16 DDR mutation carriers responded to olaparib treatment, compared to two of 33 patients in the non-DDR mutated group [56]. The promising results from this study led to the initiation of a large number of clinical trials targeting PARP by different inhibitors with or without HR gene mutation preselection in order to validate the effect, evaluate its safety profile and define the optimal timing of prescribing PARP inhibitors in mCRPC [89–92]. Interestingly, a recently published multicenter retrospective study including 23 mCRPC patients harboring DDR mutations (2 *BRCA1*, 15 *BRCA2* an 6 *ATM*) showed that men with *ATM* mutations responded inferior to PARP inhibitor treatment compared to *BRCA1/2* mutation carriers [93]. These data suggest that *ATM* mutated patients may not benefit from PARP inhibitor treatment as previously thought, and preselection of patients is importance to avoid unnecessary toxicity.

It is to be expected that due to increased use of next generation sequencing approaches, it is likely that more PCa patients with HR defects will be detected. However, the implementation and standardization of genomic testing still remains a major challenge. Besides blood-based germline mutation and biopsy based somatic mutation testing, new studies are looking into circulating tumor cells (CTC) or cell-free DNA based detection of a panel of clinically actionable genes to select eligible patients [90, 94, 95]. Moreover, the efforts made for identifying tumors with HR deficiency by using mutational signatures (HRDetect) or functional HRD tests will guide us to a more personalized cancer management approach [96–100].

#### Combination therapies

Besides PARP inhibition as monotherapy, trials have been initiated to evaluate combination of PARP inhibitors with other treatments in mCRPC patients. In view of the working mechanism of PARP inhibitors, an obvious strategy is to combine them with DNA-damaging agents, such as chemotherapy, radiotherapy and radioligand therapy (ongoing clinical trials are summarized in Table 5). Synergy with PARP inhibitors was identified in various clinical trials in other tumor types [101]. However overlapping hematological toxicities may represent a major hurdle when combining DNA-damaging agents and PARP inhibitors [102].

**Table 5:** Ongoing clinical trials with combination PARP inhibitor therapy

Strategy	Trial	Treatment	Subjects	Period	Design	Primary end point
PARP inhibitor plus AR-targeting agent	NCT02924766	Niraparib + Apalutamide or Abiraterone	mCRPC	October 2016–June 2018	A phase 1 and single group, open label study	Safety and pharmacokinetics of Niraparib
	NCT02324998	Olaparib ± Degarelix	Radical prostatectomy in men with early, localized intermediate-/high- risk PCa	December 2016–July 2018	Randomized	Determination of PARP inhibition
	NCT03395197	Talazoparib + Enzalutamide versus Enzalutamide	mCRPC with DDR defect	December 2017–May 2022	Part 1: an open-label, non-randomized, safety and PK run-in study Part 2: a randomized, double-blind, placebo-controlled, multinational study	Part 1: confirm the dose of Talazoparib Part 2: Radiographic PFS
	NCT01576172	Abiraterone ± Veliparib	mCRPC	March 2012–December 2018	A randomized gene (ETS) fusion stratified phase 2 trial	PSA response rate
	NCT03012321	Olaparib, Abiraterone, or Abiraterone + Olaparib	mCRPC with DDR defects	January 2017–January 2022	Phase 2 study randomized, open-label, multicenter	PFS
PARP inhibitor plus radioligand therapy	NCT03076203	Niraparib + Ra^223^ dichloride	mCRPC	March 2017–May 2018	Phase IB trial Single group open label	MTD
	NCT03317392	Ra^223^ dichloride + Olaparib versus Ra^223^ dichloride	mCRPC	October 2018–April 2020	A phase 1/2 study	MTD Radiographic PFS
PARP inhibitor plus VEGF inhibitor	NCT02893917	Olaparib versus Olaparib + Cediranib	mCRPC	December 2016 –December 2019	Randomized phase 2 trial	Radiographic PFS
PARP inhibitor plus immuno-therapy	NCT03431350	Niraparib + PD-1 monoclonal antibody, JNJ-63723283	mCRPC	February 2018–June 2018	A Phase 1b/2 study, Non-Randomized, Open Label Part 1 (dose selection) Part 2 (dose expansion)	Part 1: incidence of specified toxicities Part 2: objective RR and AEs
	NCT03330405	Talazoparib + Avelumab	Advanced or metastatic solid tumors (including PCa)	October 2017–March 2020	A phase 1b/2 non-randomized sequential assignment study	DLT OR
	NCT02484404	PDL-1 antibody MEDI4736 + Olaparib and/or Cediranib	Advanced recurrent PCa	June 2015–December 2019	Phase1/2 non-randomized study	Phase 1 determine the recommended phase 2 dose and the safety of combined therapy

*mCRPC*: metastatic castration-resistant prostate cancer, *RR*: response rate, *PSA*: prostate specific antigen, *PFS*: progression-free survival, *MTD*: maximum tolerated dose, *AEs*: adverse events, *DLT*: dose limiting toxicity, *OR*: overall response

Previous preclinical work offered the rationale for the potential synergy of combining AR-targeting agents with PARP inhibitors. First, blockage of AR signaling and PARP inhibition cause downregulation of the DNA repair capacity of the cells via different complementary pathways (DSB repair and SSB repair) [73, 103]. According to preclinical studies, anti-androgen treatments may induce a BRCAness phenotype, which can be targeted by PARP inhibition. Second, PARP1 has been reported to promote AR-dependent transcription and PARP inhibitors will therefore reduce AR-functioning [104]. Unfortunately, a randomized multicenter trial failed to show a significant difference in prostate-specific antigen (PSA) response rate and median progression-free survival (PFS) between patients treated with abiraterone/prednisone plus the PARP inhibitor veliparib compared to abiraterone/prednisone alone [105]. Lack of effectivity can be explained by inefficient PARP trapping by veliparib. Interestingly, another recent randomized double-blind phase 2 trial showed significantly longer PFS for mCRPC patients receiving olaparib plus abiraterone treatment than single abiraterone therapy. Although the combination strategy showed more adverse events than monotherapy, the health-related quality of life did not decline [106]. These clinical data support the preclinical results in which synergy between olaparib and AR signaling inhibitor was found, regardless of the HR status [73, 103].

Other trials are combining PARP inhibitors with vascular endothelial growth factor (VEGF) inhibitors, which function by inhibiting tumor angiogenesis. Preclinical studies showed that restriction of angiogenesis induces hypoxia, which may create a BRCAness phenotype by reducing the expression of *BRCA1* and *RAD51* [107]. The VEGF inhibitors bevacizumab and cediranib were reported to induce severe hypoxia, causing a reduction of HR capacity and increased sensitivity to PARP inhibitors [108]. Based on these data, a clinical study targeting both processes in mCRPC patients is ongoing (Table 5).

Another approach that has been explored is the use of PARP inhibitors as radiosensitizer for patients with high-risk localized PCa (radiotherapy) or with metastatic lesions (radioligand therapy). Irradiation induces cell death by the production of reactive oxygen species (ROS) as well as by direct ionization of the DNA which leads to SSBs and DSBs. PARP inhibition is predicted to enhance this effect by preventing the repair of radiation-induced SSBs. In vitro models support the idea that PARP inhibitors can enhance radiation-induced cytotoxicity [109, 110]. Similar results were also found in targeted radioligand therapy for PCa [111], suggesting targeted radiotherapy can be further optimized in combination with PARP inhibitors.

As described above, the MMR pathway has been implicated in the immunotherapy response and alterations in other DDR genes may also increase efficacy of immunotherapy [79, 112]. Therefore, several studies were started in which PARP inhibitors were combined with immunotherapy. The PARP1 inhibitor talazoparib has been found to exhibit immunoregulatory effects in a *Brca1* deficient ovarian cancer mouse model as the number of peritoneal CD8 (+) T cells and NK cells increased significantly after talazoparib treatment [113]. Furthermore, Higuchi et al. have shown that cytotoxic T-lymphocyte antigen-4 (CTLA-4) antibody synergized with PARP inhibitors therapeutically in the *Brca1* deficient ovarian cancer mouse model and support the clinical testing of this combination regimen [114]. The first clinical trial with a small cohort of patients showed that the PD-L1 inhibitor durvalumab plus olaparib in mCRPC patients has acceptable toxicity and efficacy, and the therapeutic response is superior in men with DDR abnormalities [115]. This triggered other studies to investigate whether mCRPC patients with DDR defects would benefit from this particular combination therapy. Clinical trials are ongoing to evaluate its safety, optimal dosing and efficacy (Table 5).

### Platinum-based chemotherapy

Platinum-based agents cause crosslinking of DNA, most notably interstrand crosslinks that covalently couple both DNA strands [116]. These crosslinks interfere with DNA replication and translation and induce apoptosis. Although platinum compounds have long been studied in advanced PCa patients in a large number of clinical trials, the various treatment regimens have not demonstrated a significant overall survival benefit in the overall patient population, and no treatment has received approval. Tumors with mutations in *BRCA1/2* are specifically susceptible to platinum-based chemotherapy since the interstrand crosslinks can only be adequately repaired by HR-based DNA repair. Recent clinical trials provided evidence that breast and ovarian cancer patients with *BRCA1/2* mutations are highly sensitive to platinum-based chemotherapy [99, 117, 118]. Pomerantz et al. retrospectively analyzed a single-institution cohort of mCRPC patients who received carboplatin-based chemotherapy and showed that *BRCA2* mutation carriers had a higher response rate to carboplatin-based chemotherapy than non-*BRCA2* associated patients [119]. Furthermore, a few case reports also highlighted exceptional responses to platinum-treatment in mCRPC patients with HR defects [120, 121]. With such promising results, more trials of carboplatin alone and in combination with docetaxel have been designed in advanced PCa harboring DDR aberrations (ongoing clinical trials are summarized in Table 6).

**Table 6:** Ongoing clinical trials with platinum-based chemotherapy.

Trial	Treatment	Subjects	Period	Design	Primary end point
NCT02311764C	Carboplatin	mCRPC with PTEN loss and/or DDR defect	February 2015–April 2019	A single arm, open label, phase 2 pilot study	PSA response
NCT02598895	Docetaxel + Carboplatin	mCRPC with BRCA1/2 inactivation	January 2016–June 2018	Pilot and single group assignment study	PSA response
NCT02985021	Docetaxel + Carboplatin	mCRPC with BRCA1/2, ATM inactivation	November 2016–November 2019	A phase 2 study single group assignment, open label	PSA response

*mCRPC*: metastatic castration-resistant prostate cancer, *PSA*: prostate specific antigen, *PTEN*: phosphatase and tensin homolog, *ATM*: ataxia–telangiectasia mutated serine/threonine kinase, *BRCA1/2*: Breast Cancer 1 and 2

### DNA-PKcs targeting treatment

Besides the discovery of the AR-DDR crosstalk via the key mediator DNA-PKcs, a following study has identified a new function of DNA-PKcs as a potent driver of PCa progression. Goodwin et al. found that DNA-PKcs functions as a selective modulator of transcriptional networks that induce cell migration, invasion and metastasis and suppression of DNA-PKcs inhibits tumor metastases. Moreover, DNA-PKcs levels are significantly increased in advanced disease and can be independently predictive for biochemical recurrence, poor overall survival [122]. Based on these findings, a phase I clinical trial is ongoing (NCT02833883) in which the combination of enzalutamide and DNA-PKcs inhibitor CC-115 is evaluated for treatment of mCRPC.

## Conclusion

The identification of DDR defects in mCRPC has driven the interest for further evaluation of these gene deficiencies in patient stratification. PARP inhibitors may become part of the standard care of mCRPC patients who harbor HR deficiency; however the most optimal use of PARP inhibitors alone or in combination with other treatment modalities remains to be elucidated. Given the clearly aggressive course of DDR-deficient PCa, there is an urgent need to identify these patients at an early stage where the right treatment strategy could greatly improve prognosis. The discovery that the AR may regulate DDR factors opens a new array of possible strategies to optimize treatment combinations. Future studies are needed to broaden our understanding of DDR defects and interactions between DNA repair pathways and other processes in PCa, as well as to determine how this knowledge can be used to improve diagnostic, prognostic and therapeutic approaches.
