# Correction to: Role of the DNA damage response in prostate cancer formation, progression and treatment

**DOI:** 10.1038/s41391-019-0162-1

**Published:** 2019-07-17

**Authors:** Wenhao Zhang, Dik C. van Gent, Luca Incrocci, Wytske M. van Weerden, Julie Nonnekens

**Affiliations:** 1 Department of Molecular Genetics, Erasmus MC, Rotterdam, The Netherlands; 2 Oncode Institute, Erasmus MC, Rotterdam, The Netherlands; 3 Department of Radiation Oncology, Erasmus MC Cancer Institute, Rotterdam, The Netherlands; 4 Department of Experimental Urology, Erasmus MC, Rotterdam, The Netherlands; 5 Department of Radiology and Nuclear Medicine, Erasmus MC, Rotterdam, The Netherlands

**Keywords:** Cancer therapy, Cancer genetics, Cancer genetics, Cancer therapy

**Correction to: Prostate Cancer and Prostatic Diseases**

10.1038/s41391-019-0153-2

published online 12 June 2019

This article was originally published under NPG’s License to Publish, but has now been made available under a CC BY 4.0 license. The PDF and HTML versions of the paper have been modified accordingly.

